# White Noise Speech Illusions: A Trait-Dependent Risk Marker for Psychotic Disorder?

**DOI:** 10.3389/fpsyt.2019.00676

**Published:** 2019-09-25

**Authors:** Elaine Schepers, Richel Lousberg, Sinan Guloksuz, Lotta-Katrin Pries, Philippe Delespaul, Gunter Kenis, Jurjen J. Luykx, Bochao D. Lin, Alexander L. Richards, Berna Akdede, Tolga Binbay, Vesile Altınyazar, Berna Yalınçetin, Güvem Gümüş-Akay, Burçin Cihan, Haldun Soygür, Halis Ulaş, Eylem Şahin Cankurtaran, Semra Ulusoy Kaymak, Marina M. Mihaljevic, Sanja Andric Petrovic, Tijana Mirjanic, Miguel Bernardo, Bibiana Cabrera, Julio Bobes, Pilar A. Saiz, María Paz García-Portilla, Julio Sanjuan, Eduardo J. Aguilar, José Luis Santos, Estela Jiménez-López, Manuel Arrojo, Angel Carracedo, Gonzalo López, Javier González-Peñas, Mara Parellada, Nadja P. Maric, Cem Atbaşoğlu, Alp Ucok, Köksal Alptekin, Meram Can Saka, Celso Arango, Bart P.F. Rutten, Jim van Os

**Affiliations:** ^1^Department of Psychiatry and Neuropsychology, School for Mental Health and Neuroscience, Maastricht University Medical Center, Maastricht, Netherlands; ^2^Department of Psychiatry, Yale School of Medicine, New Haven, CT, United States; ^3^Department of Psychiatry, UMC Utrecht Brain Center, University Medical Center Utrecht, Utrecht University, Utrecht, Netherlands; ^4^Department of Translational Neuroscience, UMC Utrecht Brain Center, University Medical Center Utrecht, Utrecht University, Utrecht, Netherlands; ^5^GGNet Mental Health, Apeldoorn, Netherlands; ^6^MRC Centre for Neuropsychiatric Genetics and Genomics, Division of Psychological Medicine and Clinical Neurosciences, School of Medicine, Cardiff University, Cardiff, United Kingdom; ^7^Department of Psychiatry, Dokuz Eylül University School of Medicine, Izmir, Turkey; ^8^Department of Psychiatry, Faculty of Medicine, Adnan Menderes University, Aydin, Turkey; ^9^Department of Neuroscience, Health Sciences Institute, Dokuz Eylül University, Izmir, Turkey; ^10^Ankara University Brain Research Center, Ankara, Turkey; ^11^Department of Psychology, Middle East Technical University, Ankara, Turkey; ^12^Turkish Federation of Schizophrenia Associations, Ankara, Turkey; ^13^Güven Çayyolu Healthcare Campus, Ankara, Turkey; ^14^Atatürk Research and Training Hospital Psychiatry Clinic, Ankara, Turkey; ^15^Faculty of Medicine, University of Belgrade, Belgrade, Serbia; ^16^Clinic for Psychiatry CCS, Belgrade, Serbia; ^17^Special Hospital for Psychiatric Disorders Kovin, Kovin, Serbia; ^18^Barcelona Clinic Schizophrenia Unit, Neuroscience Institute, Hospital Clinic of Barcelona, University of Barcelona, Barcelona, Spain; ^19^Institut d’Investigacions Biomèdiques August Pi I Sunyer, Barcelona, Spain; ^20^Biomedical Research Networking Centre in Mental Health (CIBERSAM), Madrid, Spain; ^21^Department of Psychiatry, School of Medicine, University of Oviedo, Oviedo, Spain; ^22^Instituto de Investigación Sanitaria del Principado de Asturias, Oviedo, Spain; ^23^Mental Health Services of Principado de Asturias, Oviedo, Spain; ^24^Department of Psychiatry, Hospital Clínico Universitario de Valencia, School of Medicine, Universidad de Valencia, Valencia, Spain; ^25^Department of Psychiatry, Hospital Virgen de la Luz, Cuenca, Spain; ^26^Health and Social Research Center, Universidad de Castilla-La Mancha, Cuenca, Spain; ^27^Department of Psychiatry, Instituto de Investigación Sanitaria, Complejo Hospitalario Universitario de Santiago de Compostela, Santiago de Compostela, Spain; ^28^Fundación Publica Galega de Medicina Xenómica (SERGAS), Universidad de Santiago de Compostela, CIBERER, Santiago de Compostela, Spain; ^29^Department of Child and Adolescent Psychiatry, Hospital General Universitario Gregorio Marañón, IiSGM, School of Medicine, Universidad Complutense, Madrid, Spain; ^30^Department of Psychiatry, School of Medicine, Ankara University, Ankara, Turkey; ^31^Department of Psychiatry, Faculty of Medicine, Istanbul University, Istanbul, Turkey; ^32^Department of Psychosis Studies, King’s College London, Institute of Psychiatry, London, United Kingdom

**Keywords:** white noise speech illusions, psychotic disorder, Community Assessment of Psychic Experiences, cognitive ability, childhood adversity, life events

## Abstract

**Introduction:** White noise speech illusions index liability for psychotic disorder in case–control comparisons. In the current study, we examined i) the rate of white noise speech illusions in siblings of patients with psychotic disorder and ii) to what degree this rate would be contingent on exposure to known environmental risk factors (childhood adversity and recent life events) and level of known endophenotypic dimensions of psychotic disorder [psychotic experiences assessed with the Community Assessment of Psychic Experiences (CAPE) scale and cognitive ability].

**Methods:** The white noise task was used as an experimental paradigm to elicit and measure speech illusions in 1,014 patients with psychotic disorders, 1,157 siblings, and 1,507 healthy participants. We examined associations between speech illusions and increasing familial risk (control -> sibling -> patient), modeled as both a linear and a categorical effect, and associations between speech illusions and level of childhood adversities and life events as well as with CAPE scores and cognitive ability scores.

**Results:** While a positive association was found between white noise speech illusions across hypothesized increasing levels of familial risk (controls -> siblings -> patients) [odds ratio (OR) linear 1.11, 95% confidence interval (CI) 1.02–1.21, *p* = 0.019], there was no evidence for a categorical association with sibling status (OR 0.93, 95% CI 0.79–1.09, *p* = 0.360). The association between speech illusions and linear familial risk was greater if scores on the CAPE positive scale were higher (*p* interaction = 0.003; OR_low CAPE positive scale_ 0.96, 95% CI 0.85–1.07; OR_high CAPE positive scale_ 1.26, 95% CI 1.09–1.46); cognitive ability was lower (*p* interaction < 0.001; OR_high cognitive ability_ 0.94, 95% CI 0.84–1.05; OR_low cognitive ability_ 1.43, 95% CI 1.23–1.68); and exposure to childhood adversity was higher (*p* interaction < 0.001; OR_low adversity_ 0.92, 95% CI 0.82–1.04; OR_high adversity_ 1.31, 95% CI 1.13–1.52). A similar, although less marked, pattern was seen for categorical patient–control and sibling–control comparisons. Exposure to recent life events did not modify the association between white noise and familial risk (*p* interaction = 0.232).

**Conclusion:** The association between white noise speech illusions and familial risk is contingent on additional evidence of endophenotypic expression and of exposure to childhood adversity. Therefore, speech illusions may represent a trait-dependent risk marker.

## Introduction

The positive symptoms of psychotic disorder are characterized by altered attribution of meaning to internal or external stimuli. It is thought that psychosis may occur across a spectrum of severity, representing a dimension of human variation extending into the general population (1, 2). Research has demonstrated that psychotic experiences—in the form of attenuated reality distortion including perceptual abnormalities and persecutory ideas—can be demonstrated in the general population (2) across the world (3). Subthreshold psychotic experiences in the general population are mostly transient in nature (4–6), but in some individuals, persistent psychotic experiences may be predictive of psychotic disorder (6, 7). According to the psychosis–proneness–persistence–impairment model, psychosis expression may become persistent and clinically relevant, depending on developmental, environmental, and cognitive factors (1, 8). Furthermore, it has been suggested that the two ends of the psychosis spectrum, from subthreshold mental variation to severe impairment, show a degree of etiological continuity. This refers to the notion that genetic (9) and environmental risk factors (10, 11) for psychotic disorder also drive variation at the level of subtle psychotic experiences in the non-ill population.

Theoretical accounts of the cognitive mechanism of hallucinations (perceptions in the absence of a stimulus) suggest that abnormal perception originates from an imbalance in top–down and bottom–up processing (12). Altered perceptions may arise when a higher priority is given to top–down processing (perceptual expectation, prior knowledge and mental imagery), at the expense of bottom–up information (sensory input) (13, 14). In this context, illusions (misinterpretations of an external stimulus) may originate from perceptual expectations associated with top–down processing (14).

Experimental illusion studies have been designed around the paradigm of hearing voices in white noise, giving rise to a speech illusion representing the tendency to attribute meaning to neutral sensory input. Hoffman and colleagues, studying a population of individuals at psychometric risk of psychotic disorder, suggested that speech illusions may signal an increased risk of psychotic disorder (13). Galdos and colleagues reported on the white noise task, showing differences between individuals with psychotic disorders and healthy participants (15), later replicated by Catalan and colleagues (16). There was a suggestion that white noise speech illusions may represent an intermediate phenotype in that a prevalence of 30% was found in patients with psychotic disorder, 14% in siblings of patients, and 9% in the general population. Hypothetically, when self-reported psychotic experiences [e.g., Community Assessment of Psychic Experiences (CAPE) positive scale] and white noise speech illusions tap into the same dimension of psychotic disorder, a positive association is expected. However, in the general population, no clear association has been observed, indicating that variations of speech illusions in the general population may not signal increased risk to develop a disorder (17, 18). A finding supporting this notion is that while risk factors for subclinical psychosis expression generally correspond with risk factors for psychotic disorder (10, 11), white noise speech illusions were not associated with either childhood adversity or life events in the general population (18). In conclusion, underlying mechanisms of white noise speech illusions may be different in patients and the general population. However, in non-ill individuals with a higher-than-average genetic risk for psychotic disorder, such as siblings of patients, white noise speech illusions may be associated with psychotic experiences as an expression of genetic risk, which may be even stronger if there is additional evidence of environmental exposure under a model of gene–environment interaction (11, 19). Furthermore, the subclass of affectively salient speech illusions (speech illusions with emotional impact) may be more strongly associated with psychotic experiences and psychosis risk. Thus, in a sample of healthy children, hallucinations during the last month were associated with white noise speech illusions that were affectively salient but not white noise speech illusions that were not affectively salient (20). Similarly, in the case–sibling–healthy participant study by Galdos and colleagues, stronger associations were apparent with affective speech illusions (15). It has been suggested that affective salience might characterize speech illusions in individuals at risk for clinical outcomes (20, 21) and may be mediated in part by cognitive alterations (22), although another study did not find evidence for this (15). Therefore, further research is required to evaluate differences in psychopathology, cognition, and affective valence of speech illusions in patients, siblings, and healthy participants.

In this report, the following hypotheses were examined. First, speech illusions were expected to be more prevalent in patients, and to a lesser degree in siblings of patients, compared to healthy participants, indicating that speech illusions may represent a familial marker for psychosis liability. Second, we hypothesized that the association between speech illusions and patient/sibling status would be trait-dependent, i.e., more pronounced in patients and relatives with greater levels of expression of known endophenotypic dimensions associated with psychosis. These included positive psychotic experiences and alterations in cognition. In addition, we hypothesized that the association between speech illusions and patient/relative status would be stronger if there was additional evidence of early environmental exposure (childhood adversity) or late environmental exposure (recent life events). Finally, investigating differences in underlying mechanisms of non-affective and affective speech illusions, we expected to find the most pronounced differences with affective speech illusions.

## Methods

The current sample was derived from Workpackage 6 of the international EU-GEI (European Network of National Schizophrenia Networks Studying Gene–Environment Interactions) project, a collaborative network studying genetic and environmental influences on the development, severity, and course of psychotic disorders (23). Workpackage 6 (GxE Vulnerability & Severity) focused on the expression of genetic and environmental liability in siblings of patients, who are thought to be at higher risk to develop psychotic disorders compared to healthy participants. In Workpackage 6, participants were collected in Spain (five centers), Turkey (three centers), and Serbia (one center). The sample consisted of 1,525 healthy comparison participants, 1,261 patients with a diagnosis of psychotic spectrum disorder (the great majority medicated patients with a diagnosis of schizophrenia or schizoaffective disorder), and 1,282 siblings of the patients. Patients were diagnosed with schizophrenia spectrum disorder according to the *Diagnostic and Statistical Manual, Fourth Edition, Text Revision* (*DSM-IV-TR*). This diagnosis was confirmed by the Operational Criteria Checklist for Psychotic and Affective Illness (24). Participants with a diagnosis of psychotic disorder due to another medical condition, a history of head injury with loss of consciousness, and an intelligence quotient <70 were excluded. The current analyses were restricted to the participants who underwent the white noise task, leaving 1,507 healthy participants, 1,014 patients, and 1,157 siblings.

In order to achieve high quality and homogeneity in clinical, experimental, and environmental assessments, standardized instruments were administered by psychiatrists, psychologists, or trained research assistants who completed mandatory on-country training sessions and online training modules including interactive interview videos and self-assessment tools (23, 25). Both on-country and online training sessions were repeated annually to maintain high inter-rater reliability throughout the 2010–2015 study enrolment period.

The EU-GEI project was approved by the medical ethics committees of all participating countries and conducted in accordance with the Declaration of Helsinki. All participants provided written informed consent.

### Cognitive Ability

A short version of the Wechsler Adult Intelligence Scale-III (WAIS-III) was used to measure cognitive ability, consisting of the Digit Symbol Coding subtest, uneven items of the Arithmetic subtest, uneven items of the Block Design subtest, and every third item of the Information subtest (26–28). The Z-score was calculated separately for each country and sex, for each test. The score for cognitive ability was calculated as the mean of the Z-scores of the different tests, expressed as a T-score (shifted and scaled to have a mean of 50 and a standard deviation of 10). In the interaction analyses, cognitive score was modeled as a binary variable, calculated around the 20^th^ percentile of the healthy participant group.

### CAPE Scale

CAPE (29, 30) is a questionnaire designed to rate self-reports of positive, negative, and depressive psychotic experiences. The questionnaire consists of 42 items: 20 items on positive psychotic experiences, 14 items on negative psychotic experiences, and 8 items on depressive feelings. Participants rated both frequency (0 = never to 4 = nearly always) and distress (1 = not distressed to 4 = very distressed) of psychotic experiences. The mean score of the frequency and distress scales was calculated for each domain. In the analyses, the frequency measure of the positive psychotic experiences was used. In the interaction analyses, CAPE score was modeled as a binary variable, calculated around the 80^th^ percentile of the healthy participant group, separately for each country.

### Childhood Adversity

Childhood adversity was assessed using the Childhood Trauma Questionnaire Short Form (CTQ), which consists of 28 items rated on a five-point Likert scale measuring five domains of maltreatment (emotional and physical neglect along with emotional, physical, and sexual abuse) (31). The psychometric characteristics of the translated versions (Spanish, Turkish, Dutch, and Serbian) of the CTQ have been comprehensively studied (32–35). Consistent with previous work in similar samples, CTQ score was modeled as a binary variable, calculated around the 80^th^ percentile of the mean score of the healthy participant group, separately for each country (36).

### Life Events

Life events were measured with an expanded version (20 items) of the Brugha List of Threatening Experiences (37, 38). Participants rated the presence of an event during the 12-month period before onset (for patients) or before the interview (for healthy participants and siblings). The sum of life events was calculated and used as a continuous measure in the analyses.

### The White Noise Task

During the white noise task, participants are exposed to three types of stimuli randomly presented across 75 fragments: 25 fragments of white noise only, 25 fragments containing white noise mixed with barely audible speech, and 25 fragments of white noise mixed with clearly audible speech. The clearly audible speech fragments had positive, negative, or neutral affective content. For example: “Sport is good for health,” “I think it is going to rain today,” or “Madrid is the capital of Spain.” Speech was adapted to each country’s native language. Each fragment had a duration of 4.3 s; the spoken sentence lasted as close as possible to 4.3 s. Sound fragments were binaurally presented through headphones. After the ending of each fragment, participants were asked to indicate what they heard by pressing on a button on the keyboard in front of them: 1, endorsed hearing speech with positive content; 2, endorsed hearing speech with negative content; 3, endorsed hearing speech with neutral content; 4, no speech heard; and 5, endorsed hearing speech but uncertain whether it was positive, negative, or neutral. The protocol was guided by the software system E-prime 1.1 (Psychology Software Tools, Pittsburgh, Pennsylvania) and took approximately 15 min. In line with the definition proposed by Catalan and colleagues (16), a speech illusion was defined broadly as a white noise fragment in which any speech was heard (option 1, 2, 3, or 5). As participants were exposed to 75 fragments, of which 25 contained white noise only, the maximum number of speech illusions was 25. First, a dichotomous speech illusion variable was calculated, with two or more speech illusions as the cutoff, as defined by Catalan and colleagues (16). Second, in order to examine the effect of affective valence, a categorical affective speech illusion variable was calculated (0 = less than two non-affective/affective speech illusions, 1 = two or more non-affective speech illusions, and 2 = two or more affective speech illusions).

### Statistical Analyses

Binary logistic regression models were applied to the dichotomous speech illusion variable. As the data were hierarchically organized (individuals nested within families), familial clustering was taken into account. A first model was run with group (healthy participants = 0, siblings = 1, and patients = 2), modeled both as a linear effect and as dummy variables, as the independent variable of main interest, corrected for age, sex, cognitive ability, and country of data collection. Second, a model was run with the interaction between group and the CAPE positive scale as an endophenotypic moderator. Similarly, a model was run with the interaction between group and cognitive ability as an endophenotypic moderator. Finally, two models were executed with speech illusions explained by the interaction between group and i) childhood adversity and ii) life events, respectively, in order to test for environmental effect modification. Interaction models were followed by calculation of stratified effects, using linear combination of terms in the model with the interaction, using the Stata lincom routine. The latter models were adjusted for the CAPE positive scale, age, sex, cognitive ability, and country of data collection.

Multinomial logistic regression models, yielding ORs, were applied to the three-level categorical affective speech illusion variable described above. The models described above for the dichotomous speech illusion variable were run again, using multinomial logistic regression with the three-level categorical affective speech illusion variable. Associations were considered significant when the two-sided p-value was <0.05; a p-value between 0.05 and 0.1 was referred to as a trend. Stata version 13 was used (39).

## Results

### Participants

An overview of selected demographic variables, familial risk, and white noise speech illusions is provided in **Table 1**. Of the 3,678 participants, 1,526 (41.5%) reported two or more speech illusions. Patients had a higher rate of speech illusions (47.2%) compared to healthy participants (41.0%) and siblings (37.1%). Inspection rates in **Table 1** suggest differences between countries in the rate of speech illusions; however, the within-country pattern of differences between groups was similar. The rate of two or more speech illusions with affective content was 14.6% in healthy participants, 8.2% in siblings, and 18.4% in patients.

**Table 1:** Summary of selected sociodemographic values.

	Healthy participants		Siblings		Patients	
Number of speech illusions (IQR)	1 (0–4)		1 (0–3)		1 (0–5)	
	Speech illusions <2 (n = 889)	Speech illusions ≥2 (n = 618)	Speech illusions <2 (n = 728)	Speech illusions ≥2 (n = 429)	Speech illusions <2 (n = 535)	Speech illusions ≥2 (n = 479)
	Mean (sd) or n (%)	Mean (sd) or n (%)	Mean (sd) or n (%)	Mean (sd) or n (%)	Mean (sd) or n (%)	Mean (sd) or n (%)
Age	33.9 (10.4)	33.8 (10.5)	33.8 (9.3)	34.1 (9.6)	33.3 (8.5)	33.3 (8.7)
Sex^a^						
Male	445 (50.1%)	314 (50.8%)	327 (44.9%)	198 (46.2%)	357 (66.7%)	324 (67.6%)
Female	442 (49.7%)	304 (49.2%)	399 (54.8%)	230 (53.6%)	178 (33.3%)	155 (32.4%)
Country						
Turkey	560 (54.4%)	470 (45.6%)	345 (56.7%)	263 (43.3%)	263 (47.4%)	292 (52.6%)
Spain	290 (67.4%)	140 (32.6%)	338 (68.4%)	156 (31.6%)	236 (57.7%)	173 (42.3%)
Serbia	39 (83.0%)	8 (17.0%)	45 (81.8%)	10 (18.2%)	36 (72.0%)	14 (28.0%)
Cognitive ability (mean)	49.9 (7.7)	50.1 (7.0)	51.2(7.7)	49.3 (8.3)	46.3 (8.1)	43.8 (8.0)
Cognitive ability (low^b^)	20.1%	17.5%	14.3%	26.3%	36.6%	50.3%
CAPE positive scale (mean)	0.28 (0.28)	0.25 (0.31)	0.22 (0.20)	0.24 (0.23)	0.59 (0.47)	0.69 (0.53)
CAPE positive scale (high^c^)	20.1%	19.9%	15.9%	21.9%	53.3%	65.6%
Childhood trauma (mean)	1.38 (0.40)	1.40 (0.39)	1.42 (0.41)	1.47 (0.43)	1.60 (0.48)	1.73 (0.54)
Childhood trauma (high^c^)	21.8%	20.1%	31.2%	35.4%	49.3%	61.6%
Life events	1.03 (1.53)	0.80 (1.57)	1.90 (1.73)	2.10 (1.76)	1.76 (1.78)	1.79 (1.75)
Current cannabis use	6.1%	5.6%	6.6%	4.8%	6.2%	4.7%

IQR, interquartile scale; sd, standard deviation; CAPE, Community Assessment of Psychic Experiences.

a Missing data in five participants.

b Binary score cutoff around the 20^th^ percentile of the distribution in the healthy participant group; 0 = high cognition score, 1 = low cognition score.

c Binary score cutoff around the 80^th^ percentile of the distribution in the healthy participant group; 0 = low CAPE/adversity score, 1 = high CAPE/adversity score.

### Associations Between Speech Illusion and Group

In models adjusted for age, sex, country, cognitive ability, and familial clustering of observations, a positive association was observed with white noise speech illusions across increasing level of familial risk (OR linear trend 1.11, 95% CI 1.02–1.21, *p* = 0.019), modeled as a linear variable. Modeling group as two dummy variables revealed that the association with white noise speech illusions was 0.93 for siblings (95% CI 0.79–1.09, *p* = 0.360) and 1.27 for patients (95% CI 1.07–1.51, *p* = 0.007).

### Psychoticism Dependence of Association Between Group and White Noise Speech Illusions

White noise speech illusions were differentially associated with the dichotomous CAPE positive scale as a function of group (OR interaction 1.32, 95% CI 1.10–1.58, *p* = 0.003). Calculation of stratified effect sizes revealed an intermediate albeit non-significant association between speech illusions and sibling status in the high CAPE positive group (OR 1.07, 95% CI 0.75–1.52, *p* = 0.713) and a larger association between speech illusions and patient status in the high CAPE positive group (OR 1.56, 95% CI 1.17–2.08, *p* = 0.003) (**Table 2**). There were no significant positive associations between speech illusions and either siblings or patients in the low CAPE positive group (**Table 2**).

**Table 2:** Interaction between group and CAPE positive scale, cognitive ability, or childhood adversity in any white noise speech illusion.

		Group	Speech illusions <2	Speech illusions ≥2	Adjusted OR (95% CI)	*P* adjusted OR	OR linear trend (95% CI)	*P* linear trend	OR interaction (95% CI)^c^	*P* interaction
CAPE positive scale^a^ × group	0	Healthy participant	704	492	1		0.96 (0.85–1.07)	0.448	1.32 (1.10–1.58)	0.003
		Sibling	589	333	0.89 (0.74–1.07)	0.222				
		Patient	230	156	0.95 (0.75–1.22)	0.696				
	1	Healthy participant	185	126	1		1.26 (1.09–1.46)	0.002		
		Sibling	139	96	1.07 (0.75–1.52)	0.713				
		Patient	305	323	1.56 (1.17–2.08)	0.003				
Cognitive ability^b^ × group	0	Healthy participant	710	510	1		0.94 (0.84–1.05)	0.255	1.53 (1.27–1.84)	<0.001
		Sibling	624	316	0.77 (0.64–0.92)	0.004				
		Patient	339	238	0.95 (0.76–1.18)	0.651				
	1	Healthy participant	179	108	1		1.43 (1.23–1.68)	<0.001		
		Sibling	104	113	2.03 (1.41–2.93)	<0.001				
		Patient	196	241	2.16 (1.57–2.97)	<0.001				
Childhood adversity^a^ × group	0	Healthy participant	695	494	1		0.92 (0.82–.04)	0.174	1.42 (1.19–1.70)	<0.001
		Sibling	501	277	0.84 (0.70–1.02)	0.087				
		Patient	271	184	0.89 (0.71–1.13)	0.349				
	1	Healthy participant	194	124	1		1.31 (1.13–.52)	<0.001		
		Sibling	227	152	1.19 (0.87–1.64)	0.272				
		Patient	264	295	1.71 (1.27–2.30)	<0.001				

OR, odds ratio; CI, confidence interval.

a Binary score cutoff around the 80^th^ percentile of the distribution in the healthy participant group; 0 = low CAPE/adversity score, 1 = high CAPE/adversity score.

b Binary cutoff around the 20^th^ percentile of the distribution in the healthy participant group; 0 = high cognition score, 1 = low cognition score.

c Average increase in risk with 1 unit change in the three-level healthy/sibling/patient variable.

### Cognition Dependence of Association Between Group and White Noise Speech Illusions

The association between white noise speech illusions and group was significantly influenced by dichotomous cognitive ability (OR interaction 1.53, 95% CI 1.27–1.84, *p* < 0.001). Stratified analyses revealed a significant association between speech illusions and sibling status in the low-cognition group (OR 2.03, 95% CI 1.41–2.93, *p* < 0.001) as well between speech illusions and patient status in the low-cognition group (OR 2.16, 95% CI 1.57–2.97, *p* < 0.001) (**Table 2**). There was a significant negative association between speech illusions and sibling status in the high-cognition group (OR 0.77, 95% CI 0.64–0.92, *p* = 0.004) and no significant association between speech illusions and patients in the high-cognition group (OR 0.95, 95% CI 0.76–1.18, *p* = 0.651) (**Table 2**).

### Environmental Moderation of Association Between Group and White Noise Speech Illusion

The association between group and white noise speech illusion was modified by dichotomously defined childhood adversity (OR interaction 1.42, 95% CI 1.19–1.70, *p* < 0.001). Stratified analyses showed a positive albeit non-significant association between speech illusions and sibling status in the group exposed to childhood adversity (OR 1.19, 95% CI 0.87–1.64, *p* = 0.272) and a larger association between speech illusions and patient status in the exposed group (OR 1.71, 95% CI 1.27–2.30, *p* < 0.001) (**Table 2**). There were no significant positive associations between speech illusions and either sibling or patient status in the low-childhood-adversity group (**Table 2**).

There was no interaction between life events in the previous 12 months and group risk in the model of speech illusions (OR 1.03, 95% CI 0.98–1.09, *p* = 0.232).

### Difference Between Non-Affective and Affective Speech Illusions

A suggestive difference was found for the association between group and non-affective white noise speech illusions (OR linear trend 1.10, 95% CI 0.99–1.21, *p* = 0.064), and a significant association was found between group and affective white noise speech illusions (OR 1.14 linear trend, 95% CI 1.00–1.29, *p* = 0.046) (**Table 3**). The interaction between group and CAPE positive scale was significant for both non-affective (OR 1.23, 95% CI 1.00 ≠ 1.51, *p* = 0.048) and affective (OR 1.57, 95% CI 1.19–2.08, *p* = 0.002) speech illusions. A significant interaction between group and cognitive ability was found in the model of affective speech illusions (OR interaction 2.44, 95% CI 1.85–3.21, *p* < 0.001), but only at trend level in the model of non-affective speech illusions (OR interaction 1.21, 95% CI 0.99–1.49, *p* = 0.069). For both non-affective (OR interaction 1.42, 95% CI 1.16–1.74, *p* = 0.001) and affective (OR interaction 1.48, 95% CI 1.13–1.93, *p* = 0.005) speech illusions, a significant interaction was found with childhood adversity (**Table 4**). No interaction was found between group and life events for either non-affective (OR interaction 1.03, 95% CI 0.98–1.09, *p* = 0.261) or affective (OR interaction 1.05, 95% CI 0.95–1.15, *p* = 0.353) speech illusions.

**Table 3:** Association between group and any non-affective and affective speech illusion.

	Number of participants	Adjusted OR (95% CI)	*P* adjusted OR	OR linear trend (95% CI)	*P* linear trend
No speech illusion^a^					
Healthy participants	889				
Siblings	728				
Patients	535				
Any non-affective speech illusion					
Healthy participants	398	1			
Sibling	334	1.07 (0.89–1.29)	0.464	1.10 (0.99–1.21)	0.064
Patients	292	1.21 (1.00–1.48)	0.056		
Any affective speech illusion					
Healthy participants	220	1			
Sibling	95	0.62 (0.47–0.81)	<0.001	1.14 (1.00–1.29)	0.046
Patients	187	1.38 (1.09–1.75)	0.007		

OR, Odds; CI, Confidence Interval.

a Reference group.

**Table 4:** Interaction between group and CAPE positive scale, cognitive ability, or childhood adversity in (non-)affective speech illusions.

		Non-affective speech illusions							Affective speech illusions					
		Group	Adjusted OR (95% CI)	*P* adjusted OR	OR linear trend (95% CI)	*P* linear trend	OR interaction (95% CI)^c^	*P* interaction	Adjusted OR (95% CI)	*P* adjusted OR	OR linear trend (95% CI)	*P* linear trend	OR interaction (95% CI)^c^	*P* interaction
CAPE positive scale^a^ × group	0	Healthy participant	1		1.01 (0.89–1.15)	0.912	1.23 (1.00–1.51)	0.048	1		0.83 (0.68–1.01)	0.065	1.57 (1.19–2.08)	0.002
		Sibling	1.03 (0.84–1.27)	0.748					0.56 (0.41–0.76)	<0.001				
		Patient	1.00 (0.76–1.32)	0.998					0.86 (0.60–1.24)	0.427				
	1	Healthy participant	1		1.24 (1.05–1.46)	0.010			1		1.31 (1.07–1.60)	0.009		
		Sibling	1.24 (0.83–1.86)	0.298					0.82 (0.49–1.36)	0.436				
		Patient	1.54 (1.10–2.16)	0.012					1.62 (1.09–2.40)	0.016				
Cognitive ability^b^ × group	0	Healthy participant	1		1.03 (0.92–1.16)	0.586	1.21 (0.99–1.49)	0.069	1		0.73 (0.61–0.88)	0.001	2.44 (1.85–3.21)	<0.001
		Sibling	0.96 (0.78–1.17)	0.670					0.38 (0.27–0.53)	<0.001				
		Patient	1.10 (0.86–1.40)	0.451					0.69 (0.49–0.97)	0.031				
	1	Healthy participant	1		1.25 (1.05–1.50)	0.013			1		1.78 (1.44–2.21)	<0.001		
		Sibling	1.81 (1.20–2.73)	0.005					2.60 (1.55–4.36)	<0.001				
		Patient	1.62 (1.12–2.34)	0.010					3.43 (2.19–5.37)	<0.001				
Childhood adversity^a^ × group	0	Healthy participant	1		0.96 (0.85–1.09)	0.552	1.42 (1.16–1.74)	0.001	1		0.84 (0.70–1.00)	0.057	1.48 (1.13–1.93)	0.005
		Sibling	0.97 (0.78–1.19)	0.746					0.54 (0.39–0.76)	<0.001				
		Patient	0.92 (0.70–1.21)	0.550					0.84 (0.60–1.19)	0.332				
	1	Healthy participant	1		1.37 (1.15–1.61)	<0.001			1		1.24 (1.00–1.53)	0.045		
		Sibling	1.58 (1.09–2.29)	0.016					0.73 (0.46–1.15)	0.178				
		Patient	1.94 (1.36–2.79)	<0.001					1.46 (0.99–2.17)	0.057				

a Binary score cutoff around the 80^th^ percentile of the distribution in the healthy participant group; 0 = low CAPE/adversity score, 1 = high CAPE/adversity score.

b Binary score cutoff around the 20^th^ percentile of the distribution in the healthy participant group; 0 = high cognition score, 1 = low cognition score.

c Average increase in risk with 1 unit change in the three-level healthy/sibling/patient variable.

Associations between speech illusions and sibling status were found for non-affective speech illusions in the low-cognition group and the high-childhood-adversity group, and for affective hallucinations in the high- (negative association) and low-cognition (positive association) groups, as well as a negative association in the low-childhood-adversity group and the low CAPE positive group.

## Discussion

This study investigated white noise speech illusions across different levels of familial risk. The analyses demonstrated that: i) white noise speech illusions were significantly associated with familial risk, modeled as a linear effect, although there was no evidence for categorical association with sibling status and ii) the association between speech illusions and familial risk, including categorical sibling status, was stronger if there was additional evidence for early environmental exposure or trait expression of psychosis proneness and cognitive alterations. The effect size of the interaction between familial risk on the one hand and cognitive ability, psychosis proneness, and environmental exposure on the other was numerically greater for affective speech illusions than for non-affective speech illusions, but these differences were small.

Two or more speech illusions were reported by 47.2% of the patients compared to 41.0% of the healthy participants and 37.1% of the siblings. These are higher than the rates reported by Galdos et al. (15), who used another definition for speech illusions (any perceived speech illusion with positive, negative, or neutral valence). The current study used the definition by Catalan et al. (16). A sensitivity analysis, however, revealed that the rate of speech illusions using the definition by Galdos et al. (40% in healthy participants, 33% in siblings, and 43% in patients) yielded a pattern of results that was similar to the results as presented above for the definition by Catalan and colleagues.

Speech illusions were not more prevalent in siblings of patients compared to healthy participants; overall, siblings even tended to have lower rates than healthy participants. However, the association between sibling status and white noise speech illusions did increase when there was additional evidence for trait expression of psychotic experiences, cognitive alterations, and childhood adversity, although this was not uniformly so, as sibling effects were most prominent for non-affective speech illusions in the *presence* of lower cognitive ability, more psychosis proneness, and environmental exposure, whereas negative sibling associations were present for affective speech illusions in the *absence* of lower cognitive ability, more psychosis proneness, and environmental exposure. In other words, in comparison to controls, siblings became more similar to patients only with respect to non-affective speech illusions in the subgroup selected for more risk traits and more exposure to risk factors. Therefore, white noise speech illusions appear to index a trait-dependent marker of risk: the power of white noise speech illusion to predict sibling and patient status is higher in the subgroup enriched with traits associated with genetic and environmental risk for the disorder.

Environmental risk factors (10, 11) for psychotic disorders have been observed to also drive variation at the level of subclinical psychotic experiences. The fact that the association between speech illusions and sibling and patient status, compared with controls, was stronger in the exposed subgroup may be suggestive of underlying gene–environment interaction. However, the results suggest that this would only apply to distal environmental exposures to life childhood adversity, but not proximal life stress. The window for crucial gene–environment interactions may be restricted to early developmental periods rather than the adult period (11).

Given that psychotic experiences and cognitive alterations are thought to reflect, in part, genetic risk for psychotic disorder (9, 40), the same underlying mechanism of gene–environment interaction may account for the dependence of patient–control and sibling–control speech illusion associations at higher levels of these traits. Thus, earlier studies have shown that in samples of the general population, no association exists between white noise speech illusions and the CAPE positive scale (17, 18). The CAPE positive scale mainly reflects alteration in delusional ideation, whereas the white noise task focuses on lower-prevalence alterations in perception. The non-significant association between speech illusions and the CAPE positive scale in the non-clinical population suggests that alterations in perception may not necessarily index alterations in ideation. It has been shown that in clinical populations and relatives of patients, alterations in perception are more strongly associated with alterations in ideation than in non-clinical populations (41). Thus, more evidence for association between white noise speech illusions and the CAPE positive scale in the trait-rich subgroup of sibling and patients, in comparison to controls, would be compatible with this observation.

The results did not suggest a uniform pattern of differentiation between affective and non-affective speech illusions in the pattern of associations between speech illusions, contrary to earlier research (15, 20). More work in this area is required.

There are some limitations. The CAPE positive scale and childhood trauma were modeled as binary variables, calculated around the 80^th^ percentile of the mean score of the healthy participant group, separately for each country. Cognitive ability was modeled as a binary variable, calculated around the 20^th^ percentile. In order to examine to what degree results were robust with regard to alternative cutoffs, sensitivity analyses were conducted with a cutoff at the 70^th^/30^th^ percentile of the healthy participant group. A similar pattern of results was found.

The white noise task and associated outcomes were only administered once. It has been suggested that persistence of psychotic experiences may be particularly predictive of psychotic disorder. Therefore, a longitudinal design allowing examination of the association between white noise speech illusions and persistence of psychotic experience may be more informative.

The study was conducted across different countries and cultures, and although the pattern of white noise distribution across patients and healthy participants was similar across countries, there were differences in base rates, which may have to do with differences in language and culture impacting the experiment. As country was adjusted for, these differences will not impact effect estimates.

## Data Availability

The datasets generated for this study are available on request to the corresponding author.

## Ethics Statement

The EU-GEI project was approved by the Medical Ethics Committees of all participating countries and conducted in accordance with the Declaration of Helsinki. All participants provided written informed consent.

## Author Contributions

All authors contributed conception and design of the study, data collection, and organization of the database. ES, RL and JV performed statistical analysis. ES wrote the first draft of the manuscript. All authors contributed to manuscript revision, read and approved the submitted version.

## Funding

Supported by the European Community’s Seventh Framework Program under grant agreement No. HEALTH-F2-2009-241909 (Project EU-GEI).

## Conflict of Interest Statement

The authors declare that the research was conducted in the absence of any commercial or financial relationships that could be construed as a potential conflict of interest.
